# P-227. Impact Of GLP-1 Receptor Agonists on Body Weight in Persons with HIV Compared to Persons Without HIV

**DOI:** 10.1093/ofid/ofaf695.449

**Published:** 2026-01-11

**Authors:** Diamond Donaldson, Marisa B Brizzi, Bailey Francis, Dimple Patel, Carl Fichtenbaum

**Affiliations:** University of Cincinnati Medical Center, Cincinnati, OH; UC Health, Cincinnati, Ohio; UC Health, Cincinnati, Ohio; UC Health, Cincinnati, Ohio; University of Cincinnati, Cincinnati, OH

## Abstract

**Background:**

Weight gain in persons with HIV (PWH) has been associated with integrase strand transfer inhibitors (INSTIs) and the mechanism of weight gain is poorly understood. Previously studied interventions, including transitioning to a non-INSTI containing regimen, have not led to adequate weight loss. Glucagon-like peptide-1 receptor agonists (GLP-1 RAs) are effective anti-obesity medications; however, data is limited on the impact of GLP-1 RA on metabolic outcomes in PWH. This study aims to evaluate the impact of GLP-1 RA on metabolic outcomes in PWH compared to persons without HIV (PWOH).

**Figure:**
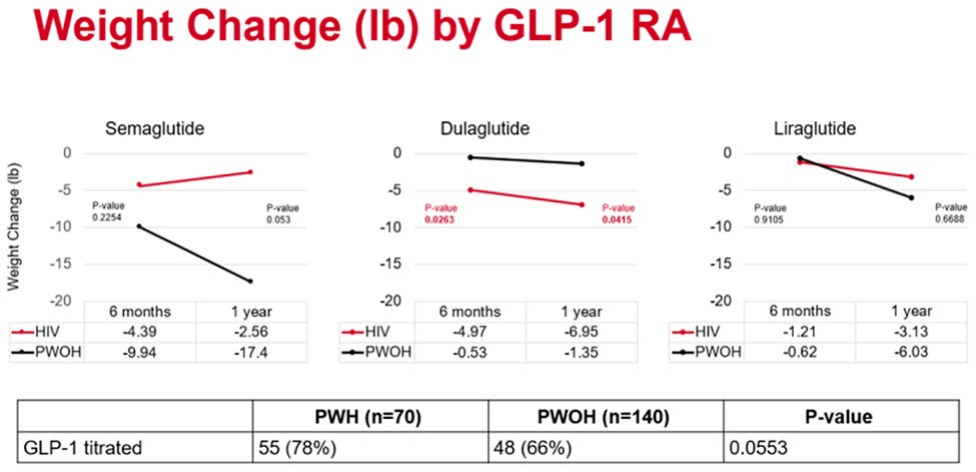

**Methods:**

This study is a retrospective cohort analysis of weight changes in PWH compared PWOH receiving GLP-1 RAs at UC Health outpatient clinics between January 1, 2017, and January 1, 2024. Outcomes were assessed by matching persons with HIV 2:1 by gender, race/ethnicity, GLP-1 RA, and dose.

**Results:**

In this analysis, 70 persons were included in the PWH group and 140 persons were included in the PWOH group. The majority of persons included were black (61%), males (57%) with a median age of 51.4 (±10.1) and 56.2 (±11.6) years in the PWH vs PWOH groups, respectively. 77% (162/210) of persons received dulaglutide, 14% (30/210) received semaglutide, and 9% (18/210) received liraglutide. The rate of persons achieving ≥5% weight loss was 27% (19/70) in the PWH group compared to 19% (26/140) in the PWOH group at 6 months (P = 0.1585) and 30% (19/64) in the PWH group and 25% (32/127) in the PWOH group at 1 year (P=0.6035). The mean change in weight was -4.98 (±12.1) lbs in the PWH group compared to -1.89 (±12.1) lbs in the PWOH group (P = 0.0825).

**Conclusion:**

In this cohort, PWH and PWOH experienced similar weight loss outcomes when receiving GLP-1 RAs. GLP-1 RAs like semaglutide and tirzepatide, that are indicated for weight loss should be studied in PWH.

**Disclosures:**

Marisa B. Brizzi, PharmD, BCPS, AAHIVP, Gilead Sciences: Advisor/Consultant|Merck: Advisor/Consultant|ViiV: Advisor/Consultant Bailey Francis, PharmD, BCIDP, AAHIVP, Merck: Honoraria Carl Fichtenbaum, MD, Gilead Sciences: Grant/Research Support|Merck: Grant/Research Support|ViiV Healthcare: Grant/Research Support

